# Literary Notes

**Published:** 1898-02

**Authors:** 


### Literary Notes.

W. B. SAUNDERS, medical publisher, announces the following books to issue during 1898: An American text-book of genito-urinary and skin diseases, by Bangs and Hardaway; Lehmann medicinische Handatlanten; Surgical diagnosis and treatment, by Macdonald; Orthopedic surgery, by Moore; Diseases of the stomach, by Van Valzah and Nisbet; The surgical complications and sequels of typhoid fever, by Keen; A compendium of insanity, by Chapin; The American year-book of medicine and surgery, by Gould.

---

THE *Philadelphia Medical Journal* made its first appearance January 1, 1898, in accordance with the announcement made in these pages in December. In size it is a large double-column quarto and in arrangement it reverses the usual order beginning with editorial matter, and then follow selections, news notes, latest literature and finally original articles. The experience of Dr. Gould as an editor and literateur justifies the belief that his new journal will assume a foremost place among the American medical weeklies.

---

THE *Microscope* has been discontinued and the *American Monthly Microscopical Journal* will be supplied to all those who wish it. The latter will be a 16-page illustrated magazine, confined to the subject of microscopy, omitting contributions to biology, publishing no long articles, but desiring abstracts, news and brief reports of interesting events. Address the publisher, Charles W. Smiley, Washington, D. C.

---

THE *Medical Age* has appeared in a new dress, which improves it in every way. It is now edited by Dr. Frederick W. Mann, who has experience in medical journal work. We offer our congratulations to this sterling semi-monthly magazine.

---

THE *International Medical Annual*, published by E. B. Treat & Co., will issue its sixteenth edition (1898) at an early day. It will contain many special articles of great interest—in addition to the regular summaries of the year's work in medicine and surgery, by thirty-eight editors, each contributing to the department with which he is especially identified. It will contain about 700 pages and will be copiously illustrated, including thirty-six full-page plates, twelve of which are finely colored. Price, $3.00 net, post free. Address the publishers, E. B. Treat & Co., 241–243 West 23d street, New York.

